# Technologies and Auditory Rehabilitation Beyond Hearing Aids: An Exploratory Systematic Review

**DOI:** 10.3390/audiolres15040080

**Published:** 2025-07-03

**Authors:** María Camila Pinzón-Díaz, Oswal Martínez-Moreno, Natalia Marcela Castellanos-Gómez, Viviana Cardona-Posada, Frank Florez-Montes, Johnatan Vallejo-Cardona, Luis Carlos Correa-Ortiz

**Affiliations:** 1 Faculty of Rehabilitation and Sports Sciences, Escuela Colombiana de Rehabilitación, Bogotá 110110, Colombia; maria.pinzon@ecr.edu.co (M.C.P.-D.); oswal.martinez@ecr.edu.co (O.M.-M.); 2 Faculty of Science and Engineering, Universidad de Manizales, Manizales 170001, Colombia; ncastellanos@umanizales.edu.co (N.M.C.-G.); vcardona@umanizales.edu.co (V.C.-P.); fflorez@umanizales.edu.co (F.F.-M.); jvallejoc@umanizales.edu.co (J.V.-C.)

**Keywords:** auditory rehabilitation, rehabilitation technology, Information and Communication Technologies, bibliographic databases

## Abstract

**Background:** Traditionally, auditory rehabilitation in people with hearing loss has sought training in auditory skills to achieve an understanding of sound messages for communication. Assistive or supportive technology is limited to hearing aids that transmit sound through the air or bone to be used by the individual, and only in recent times have technologies for rehabilitation, of high cost and difficult access, begun to be used, employed by audiology professionals. **Objective:** The objective of this study was to compile the evidence reported in the literature on the use of technology in auditory rehabilitation for the improvement of hearing skills in people with hearing loss, beyond hearing aids and cochlear implants. **Method:** A systematic review of the literature was conducted between 2018 and 2024 in PubMed, Scopus, and Web of Science databases, using as search terms Technology AND “Auditory Rehabilitation” validated in DeCS and MeSH thesauri; the PICO method was used to propose the research question, and the PRISMA strategy was used for the inclusion or exclusion of the articles to be reviewed. **Results:** In the first search, 141 documents were obtained. Subsequently, inclusion criteria, such as development with vibrotactile stimulation, Information and Communication Technologies (ICTs), among others, and exclusion criteria, such as those related to cochlear implants and air conduction hearing aids, were applied, and finally, articles related to natural language processing, and other systematic reviews were excluded so that the database was reduced to 14 documents. To this set, due to their relevance, two papers were added, for a total of sixteen analyzed. **Conclusions:** There are solutions ranging from the use of smartphones for telehealth to solutions with multiple technologies, such as the development of virtual environments with vibrotactile feedback. Hearing-impaired people and even professionals in this area of healthcare have a high level of acceptance of the use of technology in rehabilitation. Finally, this article highlights the crucial role of technology in auditory rehabilitation, with solutions that improve hearing skills and the positive acceptance of these tools by patients and audiology professionals.

## 1. Introduction

Hearing loss is a global health problem that affects 5% of the world’s population [1]. According to the World Health Organization, it is defined as a partial or total decrease in hearing ability, which can hinder communication, language development, and quality of life for those who suffer from it, in addition to being associated with mental health problems [2]. It has been shown that early intervention can “restore quality of life by eliminating, reducing, or circumventing hearing loss-induced deficits in function, activity, participation, and quality of life” [3]. In addition, the increasing prevalence of hearing loss has led to greater importance being placed on hearing loss, with hearing being recognized as playing a key role in overall well-being at all stages of life [4].

Auditory rehabilitation is a multidisciplinary approach that encompasses different strategies and therapies designed to help people with hearing loss optimize their ability to communicate and reintegrate into their listening environment [5]. These rehabilitation methods may include the use of hearing prostheses, such as hearing aids and cochlear implants, as well as speech and language therapies to improve listening comprehension and communication skills [6]. Currently, there are a variety of auditory rehabilitation methods that seek to mitigate the effects of hearing loss and improve the hearing quality of individuals. Technology has facilitated these intervention contexts; thus, the concepts of rehabilitation technologies and assistive technologies have been gaining strength and are considered basic tools that support professionals in the processes of auditory rehabilitation, with the first of them understood as those used in the rehabilitation process and the assistive ones as those that allow the user to be connected with their daily activities [7].

Traditional methods of auditory rehabilitation for people with hearing loss have focused primarily on the use of hearing prostheses, auditory training, and lip-reading [8]. Hearing aids, which include hearing aids and cochlear implants, amplify and modify sound to improve the user’s hearing [9]. Auditory training, on the other hand, consists of exercises designed to maximize the use of a person’s residual hearing and improve the ability to distinguish sounds and words in different contexts and under various noise levels [10]. Additionally, lip-reading and gestures facilitate speech understanding by supplementing limited auditory information [11]. Although these traditional methods remain valid options, technological advances continue to innovate new forms of rehabilitation for the hearing-impaired.

One of these technology-mediated approaches to auditory rehabilitation is known as vibrotactile methods, which are an alternative for the rehabilitation of hearing-impaired individuals [12]. These methods take advantage of the ability of the somatosensory system to detect vibratory stimuli on the skin [13]. They consist of the use of devices that convert acoustic signals into vibratory ones, which are transmitted to the user through oscillators placed in strategic parts of the body [14]. Vibratory stimuli encode specific characteristics of sounds, such as frequency, amplitude, or rhythm [15]. With proper training, it is possible to take advantage of these vibratory signals and obtain information about speech and other environmental sounds. Vibrotactile methods represent a novel alternative to improve auditory perception by taking advantage of the plasticity of the nervous system, thus complementing the auditory system [16]. This approach at the level of auditory rehabilitation becomes an option under the guidance of the appropriate professional since it should be based on a differential diagnosis in which the transmission by bone conduction facilitates the patient’s auditory–communicative processes.

In recent years, Information and Communication Technologies (ICTs) have revolutionized numerous fields, and auditory rehabilitation has been no exception [17]. ICT offers a wide range of innovative tools and approaches that can complement and enhance traditional methods of auditory rehabilitation. These technologies include mobile applications, online auditory training programs, assistive listening devices, and extended and alternative communication systems [18].

This systematic review article highlights the growing importance of technology in auditory rehabilitation, showing that there is a growing trend in the use of effective technological alternatives or solutions to improve hearing skills in people with hearing loss, from mobile applications to virtual environments with vibrotactile feedback. It also highlights the willingness of patients and health professionals to use these technological tools in the field of aural rehabilitation. The methodology of this article is presented below, followed by the results obtained in this review and ending with its conclusion.

## 2. Methodology

An exploratory systematic review of the content of original articles between the years 2018 and 2024 was performed. The methodology proposed in [19], subsequently reviewed in [20], was followed, consisting of developing the research question, establishing the inclusion and exclusion criteria, conducting the systematic search, selecting papers, and extracting and analyzing the results.

In the construction of the research question, the PICO strategy was used: Person/Population/Problem of Interest (P): people with hearing loss (hypoacusis); Intervention/Area of Interest (I): technologies in auditory rehabilitation; Comparison (C): other non-technological methodologies in auditory rehabilitation; (O): Improvement of auditory skills, as shown in Table 1.

Using this methodology, the following research question was developed: What is the evidence reported in the literature on the use of technology in auditory rehabilitation for the improvement of auditory skills in people with hearing loss? A search was conducted in the PubMed, Scopus, and Web of Science databases, which offer adequate coverage, accessibility, reliability, and quality for this study, using the search equation Technology AND “Auditory Rehabilitation.” The words used for this search were checked against the thesauri Descriptors of Health Sciences (DeCS) and Medical Subject Headings (MeSH).

The selection of the search strategy for the databases used in the present study was developed through the identification of key descriptors. To ensure a systematic approach, the flow chart of the Preferred Reporting Protocol for Systematic Reviews and Meta-Analyses (PRISMA) [21] was used as a fundamental guide in the formulation of this strategy. The protocol is registered in the PROSPERO platform with the number CRD420251062072 (https://www.crd.york.ac.uk/PROSPERO/view/CRD420251062072, accessed on 28 May 2025).

### 2.1. Eligibility of Documents

The criteria for the search of documents, once the equation had been defined, were the type of document: article; language: English; and subject matter: mentioning technologies used for auditory rehabilitation. Exclusion criteria were defined as articles that mentioned cochlear implants, conventional hearing aids, or similar technologies, as well as other systematic reviews. Searches were limited to articles published between 2018 and 2024, and 141 articles were obtained.

### 2.2. Selection of Documents

**Figure 1:**
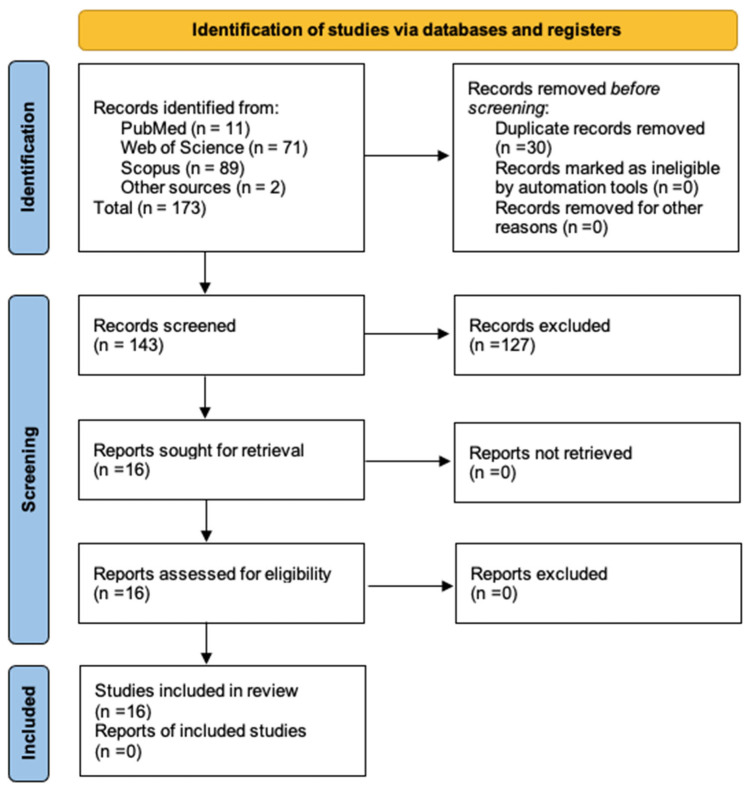
PRISMA diagram.

Based on the results of each database consulted, the reference, title, abstract, and keywords were extracted from each one. As for the selection of the documents, a two-stage process was carried out. In the first stage, filtering of the documents after the elimination of duplicates was performed by reading the title and the summary of the results. In the second stage, the documents were selected by reading the full text, leaving a total of 14 documents for review. A total of 127 were discarded because, although the main topic was auditory rehabilitation, these did not involve technologies other than hearing aids used by people with hearing loss, established as an exclusion criterion, or its subject matter was not relevant to this study. Finally, two additional papers were included because of their relevance. Within the emerging themes that group the various documents, three categories are structured: Telehealth and ICT Applications, Frequency-Modulated Systems, and Vibrotactile Stimulators. The whole process can be seen in Figure 1.

## 3. Results

Technology has undoubtedly been used in auditory rehabilitation, as evidenced by the documents reviewed. The table below presents a list of the 16 reviewed documents, showing their authors, year, country of origin, objective, outcome, and category of analysis.

Solutions vary from the use of smartphones as a low-cost resource for monitoring and controlling people undergoing treatment to virtual reality environments with vibrotactile feedback to better capture the information sent in sound signals placed in different scenarios. Three areas of analysis are presented below: telehealth and ICT applications, frequency-modulated systems, and vibrotactile stimulators.

### 3.1. Telehealth and ICT Applications

ICTs play a key role in hearing recovery by improving access to rehabilitation services and facilitating personalized care [22]. Smartphone applications allow for real-time monitoring of hearing aid use and provide valuable data to healthcare professionals, which encourages shared decision-making and goal-setting for each individual in the rehabilitation process [23].

Telehealth significantly improves auditory rehabilitation outcomes by providing flexible and timely access to care and facilitating better self-management for those who are cared for. Virtual consultations allow for frequent monitoring of the use of hearing aids or other technologies through data logging, which helps to effectively troubleshoot problems and increase usage rates [23]. In addition, telehealth allows individuals to prepare for consultations by submitting questionnaires in advance, allowing therapists to focus on treatment options rather than data collection during consultations [24].

Computer-based auditory training platforms have been shown to provide at least equivalent speech comprehension outcomes compared to traditional face-to-face therapy, demonstrating both clinical efficacy and cost effectiveness. There is evidence that such applications are useful for improving listening skills and reducing dependence on face-to-face therapy, especially for cochlear implant users [25]. These technologies not only promote self-management and empower patients but also improve the overall quality of life for people with hearing loss by addressing both auditory and nonauditory effects [22]. Therefore, the integration of technology in hearing recovery is critical to achieve successful outcomes.

Research indicates that virtual and augmented reality technologies can be used effectively in auditory training for children with cochlear implants and hearing aids, improving their auditory skills through engaging and interactive experiences [26]. In addition, auditory rehabilitation, which encompasses various services aimed at mitigating the impact of hearing loss, can benefit from virtual reality by providing immersive training environments that foster better learning outcomes [25]. Overall, virtual, augmented, and mixed realities present a novel approach to make aural rehabilitation more effective and user-friendly.

Finally, mobile apps for hearing therapy offer several important benefits, especially for older adults and families who are hearing-impaired. They provide convenient access to educational and support resources, which improves hearing aid self-management and rehabilitation processes [27]. The use of apps can facilitate real-time monitoring and data logging, enabling audiology professionals to provide timely support and address challenges effectively [25]. Apps such as HeRO have been developed to tailor auditory training to individual needs, promoting user engagement and ease of use among elderly users [27]. Overall, these mobile solutions not only improve accessibility and flexibility but also encourage better compliance with auditory therapy recommendations, while novel approaches such as the one put forward in [28] evidence a promising future in the development and validation of technologies in aural rehabilitation.

### 3.2. Frequency-Modulated (FM) Systems

Frequency Modulation (FM)-based assistive listening systems are technologies designed to improve sound reception for users with hearing loss, especially in noisy or complex listening environments [29]. These systems use external microphones that pick up acoustic signals, usually at a considerable distance from the hearing aid, and transmit the audio directly to the patient’s ear. This process significantly reduces the impact of ambient reverberation and background noise, providing the user with a clearer and more focused sound signal. As a result, the cognitive effort required to process sound in acoustically challenging environments is decreased, which, in turn, reduces auditory exhaustion [30].

Several studies have shown that FM systems can improve the signal-to-noise ratio (SNR) by up to 25 dB, which represents a considerable increase in speech clarity in environments with background noise [31]. These systems are especially useful in situations where competing noise is prominent or when the user needs greater concentration and attention on the auditory stimulus, such as in educational or work environments. In addition, FM systems have been documented to be “devices that help hearing-impaired people reduce the effects of ambient noise” [32].

Although their use is more common in educational or work environments, where the need for clear speech perception is crucial, the use of FM systems in home environments is less widespread [33]. However, in these environments, there may also be high noise levels that negatively affect speech perception, suggesting a potential benefit of these systems in everyday situations that require effective communication. In [34], it is stated that people using FM systems report improved speech perception in environments with background noise, underscoring the importance of these devices in the daily lives of those facing hearing challenges. However, these devices must undergo rigorous measurement and testing to confirm their effectiveness. The acceptance and success of an FM system in the marketplace depend largely on the functional and clinical tests performed, thus ensuring its efficacy, safety, and reliability [35].

In addition to clinical testing, it is important that hearing healthcare professionals provide adequate and personalized guidance to users on the use of these devices. Training in the handling of FM systems, together with accurate fitting, can maximize the benefits perceived by the user, ensuring successful integration into their daily life [36].

### 3.3. Vibrotactile Stimulators

Hearing is a process in which peripheral structures intervene, whose objective is to carry out the transit of auditory stimuli until they reach the central auditory processing, which is the use given to the information perceived from the structures that carry out the transit of sounds [37]. This entry of sounds, from a biological and physical approach, can occur through two routes: air and bone. The conduction of sound via bone is established as the transmission of acoustic waves from a gaseous medium to the structures of the inner ear through the cranial bones [38]. The variations that can occur in acoustic phenomena are defined by the ease with which a sound is conducted through solid media rather than in liquid and gaseous media [39].

Since 1960, there has been growing interest in studying bone conduction in the auditory process, evidenced by approaches such as that of Guberina, which supports the processes of auditory rehabilitation, specifically the verbo-tonal method, on the principle of bone conduction [40]. From acoustic physics, this type of conduction has more vibration at low frequencies, i.e., more acoustic power. Technological immersion and new advances in auditory rehabilitation processes have considered this principle the basis for the generation of technology that favors auditory–communicative processes in individuals [41].

These systems have demonstrated significant gains in such complex tasks as speech processing, specifically in high-demand environments such as noise. Studies report a significant improvement of 6.3% in speech understanding in noise, using vibrotactile impulses, which considered syllable timing and other speech properties. This type of transmission employs existing neural pathways, which generate multisensory effects, facilitating the processing of acoustic signals [42].

Some studies focus on the location of the device that will make the vibrotactile feedback, as is the case of the study by Adler et al. [41], whose device-ring or bracelet was placed in the person’s hand and they make a frequency adjustment. A similar process happens with the creation of a glove that allows for sending stimulating signals that transmit not only acoustic stimuli but also emotional information; these signals are accompanied by videos and images not associated with dramatic actions, resulting in the activation of areas of the auditory cortex (superior temporal cortex, insula, auditory cortex, inferior frontal gyrus) and of the emotional domain, with music as the main element to be transmitted in the process of acoustic stimulation [43].

Another study that supports the creation of tools based on vibrotactile stimulation was developed by Tufatulin et al. [40], who established that patients who received vibrostimulation in a program created in an aquatic environment (swimming pool), before the adaptation of hearing aids or cochlear implants, presented better results than children who had not received this previous treatment. It was concluded that these approaches reduce consultation times and intervention sessions; this type of intervention favors severe frequencies and has been considered a more effective means of early intervention in children [40].

This type of product has not only shown results in international contexts, as, in Colombia, different professionals have shown interest in this topic. The research developed by students of sound engineering at San Buenaventura University in 2019 had, as a product, a sensory feedback system through bone conduction for people with visual impairment, facilitating the processes of mobility by generating an auditory alert to changes in the color of the traffic light, releasing the external auditory canal without generating any barrier time that decreases the intensity of auditory stimuli [44].
**Author, Year, and Country****Aim****Result****Category of Analysis**Timmer, Launer & Hickson, 2020, Australia [22]To describe research that has applied mobile applications at various stages of the adult rehabilitation process.Mobile apps can provide practical information during the early stages of the patient experience and support for hearing aid management in the later stages. The usability of the app was rated positively by participants.Telehealth and ICT ApplicationsVölter, Stöckmann, Schirmer, & Dazert, 2021, Germany [25]To evaluate the feasibility of a new platform for teletherapy auditory rehabilitation in adults and to compare the clinical outcomes and economic benefits of this platform with those derived from conventional face-to-face rehabilitation settings.The proposed teletherapy approach to auditory rehabilitation allows for good clinical outcomes while saving time for users and healthcare personnel.Telehealth and ICT ApplicationsHatzigiannakoglou & Okalidou, 2019, Greece [26]To describe an auditory training software and demonstrate its usability in children.The children with hearing loss were able to use the game successfully. This positive outcome supports the use of virtual reality and immersive technology as auditory training tools.Telehealth and ICT ApplicationsKwak, Kim, You & Han, 2020, Republic of Korea [27]To develop a mobile healthcare application for elderly who suspect or know they have hearing loss, called Hearing Rehabilitation for Older Adults (HeRO).There is a clear intention to use the healthcare application because seniors readily accept it and find it easy to manipulate.Telehealth and ICT ApplicationsPaccola, Costa- Filho, & Jacob, 2021, Brazil [29]To propose and validate a protocol for the electroacoustic verification of the FM system coupled to the bone conduction hearing aid.The transparency criterion was achieved in all evaluated devices, but in some combinations, it was necessary to adjust the gain of the FM receiver above the manufacturer’s default setting. The proposed protocol was effective for electroacoustic verification of the FM system coupled to the bone conduction hearing aid.Frequency-Modulated (FM) SystemsOosthuizen, Picou, Pottas, Myburgh, & Swanepoel, 2021, Republic of South Africa [30]To evaluate the effects of two intervention options, the Remote Microphone System (RMS) and the Contralateral Routing of Signal (CROS) system, in school-aged children with unilaterally limited hearing.The RMS could be a beneficial technology option in classrooms for children with unilaterally limited hearing.Frequency-Modulated (FM) SystemsChen, Wang, Dong, Fu, Wang, & Wang, 2021, China [31]To evaluate improvements in speech recognition capability in noisy environments as measured by the signal-to-noise ratio using wireless remote microphone-based technologies.Technologies incorporating a wireless remote microphone can significantly improve speech recognition performance in challenging listening environments for Mandarin-speaking hearing aid users in China.Frequency-Modulated (FM) SystemsGabova, Meier, & Tavel, 2024, Czech Republic [33]To explore parents’ experiences with Remote Microphone Systems (RMSs) for their children with hearing loss and to determine the advantages and disadvantages perceived by parents.Parents listed the advantages of RMS for their children, themselves and other caregivers as improved hearing and comprehension, a life more like that of peers without hearing loss, road safety, reduced fatigue, vocabulary acquisition, and improved school performance. Some limitations were identified, such as low benefits, technical problems, and reluctance to use the device by children or teachers.Frequency-Modulated (FM) SystemsDhanjal & Singh, 2019, India [34]To analyze various assistive technology tools and techniques for the hearing-impaired.This article contributes to providing a common platform for research conducted by various researchers in multidimensional areas of assistive devices, including commercially viable devices available on the market.Frequency-Modulated (FM) SystemsScarinci, Nickbakht, Timmer, Ekberg, Cheng, & Hickson, 2022, Australia [36]To explore the perceptions and experiences of hearing-impaired adults, their significant others, and healthcare personnel regarding the use and provision of Wireless Microphone Systems (WMSs).The study results highlight the complexity of providing and using WMSs with hearing-impaired adults. Both significant others and healthcare professionals reported that, with the right experience, expectations, training, and support, wireless microphone systems can make a significant difference in listening and communication in different situations.Frequency-Modulated (FM) SystemsPeralta & Piccolini 2022, Argentina [39]To implement a system to measure the speed of sound propagation in water directly.The implemented device was able to measure both speed and depth, but with a higher accuracy error than expected.Vibrotactile StimulatorsTufatulin, Koroleva, Artyushkin & Yanov, 2021, Russia [40]To determine the limits of perception of underwater vibrotactile stimuli and to measure the effect of vibrostimulation training on auditory rehabilitation in young children.Underwater vibrostimulation is a promising method of early auditory rehabilitation that could be recommended for implementation in pediatric audiology centers.Vibrotactile StimulatorsAdler, Sanchez, Martini, Vartabedian, Zazzali, & Quintero-Rincon, 2020, Argentina [41]To study the vibrations produced by a sound transmitted by bone conduction between a mobile phone and the hand, analyzed with the DSP Logger MX equipment.This study clarified both the transmission quality, and the attenuation caused by the hand, facilitating the design of a future bone conduction communication device. With the results obtained, it is concluded that, in terms of sound transmission, a ring design would have lower power loss than a bracelet design since it decreases with distance.Vibrotactile StimulatorsGuilleminot & Reichenbach, 2022, USA [42]To demonstrate that the presentation of vibrotactile signals at syllabic rate can improve speech understanding in the presence of background noise.Evidence is provided that this multisensory improvement in speech understanding reflects multisensory integration of auditory and tactile information in the auditory cortex.Vibrotactile StimulatorsLucia, Revuelta, Garcia, Ruiz, Vergaz, Cerdán, & Ortiz, 2020, Spain [43]To explore an alternative method that uses vibrotactile stimulation as a possible channel to convey the emotional information contained in the audio section of a video and thus elicit a greater emotional reaction in hearing-impaired individuals.The main result is that it shows that vibrotactile stimuli can generate activation of the cortex while watching a video, similar to sound.Vibrotactile StimulatorsAbril-Linares, & Romero-Tellez, 2019, Colombia [44]To implement an auditory sensory feedback system using bone conduction to deliver audio alerts about the status of a traffic light.The individuals evaluated reported that the messages received through bone conduction were clear and concise.Vibrotactile Stimulators


## 4. Conclusions

The integration of information and communication technologies, such as telehealth, mobile applications, and virtual and augmented realities, has transformed aural rehabilitation, improving access to services, and personalization of treatment and clinical outcomes. These tools not only facilitate self-management and empower patients but also optimize the efficiency of consultations and promote greater adherence to therapy, standing out as key elements for success in hearing recovery.

The technological resources used for auditory rehabilitation have been shown in two lines of approach, the first being defined by open access technology, such as telehealth, mobile applications, and virtual and augmented realities [34], which reduces consultation times and focuses on one of the most significant difficulties of these approaches in health systems: to be able to perform an effective follow-up [22]. For the professional, this process has become a challenge, considering barriers such as distance and timeliness in the consultation. Thus, the use of applications is consolidated as a fundamental tool for both the professional and the user, who today becomes a key player in the process due to the ease of use generated by these tools to check the status of hearing devices and access training programs designed for their hearing impairment.

The second refers to devices such as frequency-modulated systems and vibrotactile stimulators, which present the user with significant advances in speech perception in adverse conditions and in central auditory processing [34]. These innovations stand out for their ability to address specific needs in complex acoustic environments, reducing listening effort and optimizing intervention results [43], in addition to better results in signal processing, significantly improving speech understanding performance in patients with hearing loss.

It is necessary to continue developing research and innovations in hearing rehabilitation technology. These processes should be led by professionals with competencies in aural rehabilitation, as well as by experts in electronics, engineering, rehabilitation, and technology, through the development and improvement of new devices, to respond to the auditory–communicative needs of both individuals and populations.

## Figures and Tables

**Table 1:** PICO criteria.

P	People with hearing loss (hypoacusis)
I	Technologies in auditory rehabilitation
C	Other non-technological methodologies in auditory rehabilitation
O	Improvement of auditory skills

## Data Availability

No new data were created or analyzed in this study. Data sharing is not applicable.
